# Plasma Autoantibodies against Heat Shock Protein 70, Enolase 1 and Ribonuclease/Angiogenin Inhibitor 1 as Potential Biomarkers for Cholangiocarcinoma

**DOI:** 10.1371/journal.pone.0103259

**Published:** 2014-07-24

**Authors:** Rucksak Rucksaken, Chawalit Pairojkul, Porntip Pinlaor, Narong Khuntikeo, Sittiruk Roytrakul, Carlo Selmi, Somchai Pinlaor

**Affiliations:** 1 Department of Parasitology, Faculty of Medicine, Khon Kaen University, Khon Kaen, Thailand; 2 Department of Pathology, Faculty of Medicine, Khon Kaen University, Khon Kaen, Thailand; 3 Centre for Research and Development in Medical Diagnostic Laboratory, Faculty of Associated Medical Sciences, Khon Kaen University, Khon Kaen, Thailand; 4 Department of Surgery, Faculty of Medicine, Khon Kaen University, Khon Kaen, Thailand; 5 Liver Fluke and Cholangiocarcinoma Research Center, Faculty of Medicine, Khon Kaen University, Khon Kaen, Thailand; 6 Proteomics Research Laboratory, Genome Institute, National Center for Genetic Engineering and Biotechnology, Pathumthani, Thailand; 7 Division of Rheumatology and Clinical Immunology, Humanitas Clinical and Research Center, Rozzano, Milan, Italy; 8 BIOMETRA Department, University of Milan, Italy; Technische Universitaet Muenchen, Germany

## Abstract

The diagnosis of cholangiocarcinoma (CCA) is often challenging, leading to poor prognosis. CCA arises via chronic inflammation which may be associated with autoantibodies production. This study aims to identify IgG antibodies directed at self-proteins and tumor-associated antigens. Proteins derived from immortalized cholangiocyte cell line (MMNK1) and CCA cell lines (M055, M214 and M139) were separated using 2-dimensional electrophoresis and incubated with pooled plasma of patients with CCA and non-neoplastic controls by immunoblotting. Twenty five immunoreactive spots against all cell lines-derived proteins were observed on stained gels and studied by LC-MS/MS. Among these, heat shock protein 70 (HSP70), enolase 1 (ENO1) and ribonuclease/angiogenin inhibitor 1 (RNH1) obtained the highest matching scores and were thus selected for further validation. Western blot revealed immunoreactivity against HSP70 and RNH1 in the majority of CCA cases and weakly in healthy individuals. Further, ELISA showed that plasma HSP70 autoantibody level in CCA was significantly capable to discriminate CCA from healthy individuals with an area under the receiver operating characteristic curve of 0.9158 (cut-off 0.2630, 93.55% sensitivity and 73.91% specificity). Plasma levels of IgG autoantibodies against HSP70 were correlated with progression from healthy individuals to cholangitis to CCA (r = 0.679, *P*<0.001). In addition, circulating ENO1 and RNH1 autoantibodies levels were also significantly higher in cholangitis and CCA compared to healthy controls (*P*<0.05). Moreover, the combinations of HSP70, ENO1 or RNH1 autoantibodies positivity rates improved specificity to over 78%. In conclusion, plasma IgG autoantibodies against HSP70, ENO1 and RNH1 may represent new diagnostic markers for CCA.

## Introduction

Cholangiocarcinoma (CCA) is an adenocarcinoma originating from bile duct epithelial cells with highest incidence rates in Northeastern Thailand [1]. In this area, CCA is associated with the liver fluke, *Opisthorchis viverrini* infection through chronic inflammation of the bile ducts [2], [3] via reactive oxygen and nitrogen species [4], [5] and reactivity against the infection [6], [7]. Ultrasonography currently represents the most sensitive tool for hepatobiliary cancer detection but this is often difficult to discriminate from cholangitis [8] and, as a result, diagnosis is frequently delayed with dramatic effects on the outcome [9], [10]. Serum carcinoembryonic antigen (CEA) and carbohydrate antigen 19-9 (CA 19-9) are commonly used when CCA is suspected but their sensitivity and specificity are widely variable [11]–[13] and, as a result, there is an urgent need for new noninvasive biomarkers.

Autoantibodies against self- or tumor-associated antigens (TAAs) represent promising cancer biomarkers [14]–[18]. In several cases, autoantibodies correlate with disease stage [19]–[21] and persist longer and at higher levels than the targeted protein [22]. Mechanisms underlying the production of autoantibodies against the bile ducts remain enigmatic [23], possibly relying on the link with chronic inflammation [24] with oxidative stress causing the appearance of neoepitopes [25]. Moreover, it is now largely established that immune response to TAAs may be affected by many mechanisms including mutated [26], misfolded [27], overexpressed [28], aberrantly degraded [29], glycosylated [30] and ectopically expressed [31] tumor proteins.

Based on these observations, we sought for plasma autoantibodies in patients with fluke-associated CCA and identified immunoreactivity against heat shock protein 70 (HSP70), enolase 1 (ENO1) and ribonuclease/angiogenin inhibitor 1 (RNH1) proteins as potential biomarkers for CCA.

## Methods

### Subjects

Plasma samples were obtained from 66 subjects and divided into 3 groups including healthy controls (n = 23, 17 healthy subjects and 6 abnormal blood vessels subjects without hepatobiliary tract abnormality, mean age 53.4±8 years), patients with cholangitis (bacterial cholangitis and cholelithiasis, n = 12, mean age 58.7±10 years) and CCA (of tubular and papillary types, n = 31, mean age 56±9 years). Ten ml of peripheral blood were obtained by sterile venipuncture and collected in tubes containing EDTA. Blood was centrifuged at 3,000 g for 15 min at 4°C. Plasma samples were stored at –80°C until analyzed. The study protocol was approved by the Human Research Ethics Committee, Khon Kaen University, Thailand (HE561290) and a written informed consent was obtained from all subjects (HE521209). Patients with liver cancer were undergoing hepatectomy at Srinagarind Hospital, Faculty of Medicine, Khon Kaen University, Thailand. Diagnosis of cholangitis and CCA was based on clinical, radiological, laboratory criteria and confirmed by liver biopsy. Healthy individuals were age- and sex-matched to patients with liver cancer and manifested no *O. viverrini* eggs in stool, normal urinalysis and normal hepatobiliary tract assessed at ultrasonography.

### Cell lines and cell culture

An immortal cholangiocyte (MMNK1) was established as previously described [32]. Human CCA (moderately differentiate type (M055 and M214) and squamous cell carcinoma type (M139) were developed from Thai patients and written informed consents were obtained from all subjects. CCA cell lines were established and characterized as described previously [33]. CCA cell lines were kindly provided by Prof. Banchop Sripa, Department of Pathology, Faculty of Medicine, Khon Kaen University, Thailand. All cell lines were cultured in HAM’s F-12 medium supplemented with 10% heat-inactivated fetal bovine serum (FBS), 2 mmol/L glutamine, 15 mmol/L HEPES and 14 mmol/L sodium bicarbonate, 100 U/ml penicillin G and 100 U/ml streptomycin. Cells were maintained at 37°C under a 5% humidified CO_2_ incubator. Cell culture materials (medium, FBS and antibiotics) were purchased from Gibco Invitrogen (Auckland, New Zealand).

### Two-dimensional (2D) gel electrophoresis and western blotting

Pellets from MMNK1 and CCA cell lines (M055, M214 and M139) were lysed by freeze-thaw and added with 150 µl of sample preparation solution (8 M urea, 2 M thiourea, 4% (wt/vol) CHAPS (3-[(3-cholamidopropyl)-dimethylammonio]-1-propanesulfonate), 2% (vol/vol) immobilized pH gradient (IPG) buffer (pH 3 to 10), 40 mM dithiothreitol (DTT) and protease inhibitors cocktail) (GE Healthcare, Piscataway, NJ, USA). Samples were then vortexed, incubated at 4°C for 1 h and centrifuged at 12,000 rpm for 30 min at 4°C. Supernatant were collected and protein concentration was measured using Bradford assay (Bio-Rad Laboratories, Hercules, CA, USA) according to the manufacturer’s protocol. Proteins (150 ug) from each cell lines were mixed in the sample loading buffer with 0.5% IPG buffer, pI 3–10 (GE Healthcare, Tokyo, Japan) and isoelectric focusing using the Immobiline DryStrip, pH 3–10, 7 cm (GE Healthcare) using Ettan IPGphor II (GE Healthcare) followed by 12% SDS-PAGE. Proteins in the gel were transferred to a polyvinylidene difluoride membrane (PVDF membrane, Amersham Bioscience, Piscataway, NJ, USA) and blocked with 5% skim milk, 0.1% Tween 20 in phosphate buffer saline (PBS) for 1 h at room temperature, then incubated with the 1∶2000 diluted pooled plasma CCA samples (n = 10) in 5% skim milk, 0.1% Tween 20 in 1× PBS (PBST) for 1 h at room temperature. After washing, the membrane was incubated with anti-human IgG HRP conjugate dilution 1:15,000 (Santa Cruz Biotechnology, Santa Cruz, CA, USA) for 1 h at room temperature. The membrane was developed using ECL solution (GE Healthcare). The parallel 2D gels and PVDF membranes were also stained with coomassie brilliant blue (CBB) in order to match spots with protein in CBB gels.

### Tryptic digestion and protein identification by mass spectrometry

Following comparisons with western blot membranes, corresponding spots on CBB staining gels were matched and excised. Excised spots were prepared using tryptic digestion for LC-MS/MS. In brief, the gel spot containing the protein was destained, reduced and alkylated, then digested with 10 ng/µl trypsin in 50% acetonitrile (ACN)/10 mM ammonium bicarbonate (Promega, Madison, WI, USA) followed by incubation at room temperature for 20 min. To keep the gels immersed throughout digestion, 20 ml of 30% ACN was added and incubated at 37°C overnight. To extract peptide digestion products, 30 ml of 50% ACN in 0.1% formic acid was added into the gels and incubated at room temperature for 10 min in a shaker. Peptides extracted were collected, dried by vacuum centrifuge and kept at −80°C for further mass spectrometric analysis. Nanoscale LC separation of tryptic peptides was performed with a NanoAcquity system (Waters Corp., Milford, MA, USA) equipped with a Symmetry C18 (5 µm, 180-µm×20-mm) Trap column and a BEH130 C18 (1.7 µm, 100-µm×100-mm) analytical reversed phase column (Waters). Samples were initially transferred with an aqueous 0.1% formic acid solution to the trap column with a flow rate of 15 µl/min for 1 min. Mobile phase A was 0.1% formic acid in water and mobile phase B was 0.1% formic acid in acetonitrile. The peptides were separated with a gradient of 15–50% mobile phase B over 15 min at a flow rate of 600 nl/min followed by a 3-min rinse with 80% of mobile phase B. The column temperature was maintained at 35°C. The lock mass was delivered from the auxiliary pump of the NanoAcquity pump with a constant flow rate of 500 nl/min at a concentration of 200 fmol/µl of [Glu1]fibrinopeptide B to the reference sprayer of the NanoLockSpray source of the mass spectrometer. All samples were analyzed in one sitting. Analysis of tryptic peptides was performed using a SYNAPT-HDMS mass spectrometer (Waters). For all measurements, the mass spectrometer was operated in the V-mode of analysis with a resolution of at least 10,000 full-width half-maximum. All analyses were performed using positive nanoelectrospray ion mode. The time-of-flight analyzer of the mass spectrometer was externally calibrated with [Glu1]fibrinopeptide B from m/z 50 to 1600 with acquisition lock mass corrected using the monoisotopic mass of the doubly charged precursor of [Glu1]fibrinopeptide B. The reference sprayer was sampled with a frequency of 20 sec. Accurate mass LC-MS data were acquired with data direct acquisition mode. The energy of trap was set at collision energy of 6 V. In transfer collision energy control, low energy was set at 4 V. The quadrupole mass analyzer was adjusted such that ions from m/z 300 to 1800 were efficiently transmitted. The MS/MS survey was over range 50 to 1990 Da and scan time was 0.5 sec. The spectral data were generated in a micromass file (PKL) support for MS/MS Ion Search using the MASCOT engine program (Matrix Science, London, UK). MS data were searched against the NCBI database for human proteins and the search was carried out with the following parameters: MS/MS Ion Search using trypsin enzyme, carbamidomethylated cysteine as fixed modifications; oxidation of methionine residues as a variable modification used, monoisotopic mass values and unrestricted protein mass. Peptide mass tolerance was ±1.2 Da, fragment mass tolerance was ±0.6 Da and maximum of zero to one missed cleavage. ESI-QUAD-TOF was the instrument type. All identified proteins exhibited a Mascot score greater than 66 were considered statistically significant (*P*<0.05).

### Plasma autoantibodies by western blotting

To validate autoantibodies against HSP70, ENO1 and RNH1 as CCA markers, 0.2 µg of recombinant human full-length HSP70 (Sigma-Aldrich, St. Louis, MO, USA), ENO1 and RNH1 (Abnova, Walnut, CA, USA) proteins were separated on 10% SDS-PAGE gel and transferred to a PVDF membrane (Amersham Bioscience, Piscataway, NJ, USA) membrane (Amersham Bioscience, Piscataway, NJ, USA) for 1 h at 0.35A. The membranes were cut and incubated with individual plasma samples (dilution 1∶250) of representative cases from healthy controls (n = 5) or patients with CCA (n = 7). Then, the membranes were incubated with anti-human IgG HRP conjugated, dilution 1:2,500 (Santa Cruz Biotechnology) diluted in 2% skim milk/PBST for 1 h at room temperature. The membranes were developed using ECL solution (GE Healthcare). Pooled healthy plasma (negative control) and pooled plasma from patients with CCA (positive control) were performed in the same run.

### Plasma autoantibodies by enzyme-linked immunosorbent assay

Plasma anti-HSP70, ENO1 and RNH1 IgG autoantibodies were measured by indirect ELISA in 66 plasma samples from healthy individuals (n = 23), patients with cholangitis (n  = 12) and CCA (n = 31). The 96-well MaxiSorp immunoplates (Nunc, Roskilde, Denmark) were coated with 100 µl of recombinant human full-length HSP70, ENO1 or RNH1 (each, 1 µg/ml in 1× PBS) at 4°C overnight and washed once with 1× PBST. The plates were blocked with 100 µl of 5% skim milk for 2 h at room temperature and washed two times with 1× PBST. Then, the reactions were added with 100 µl of each 250-fold diluted plasma samples in dilution buffer (1× PBS pH 7.4, 5% skim milk, 0.05% Tween 20) and incubated for 1 h at 37°C. After washing three times with 1× PBST, 100 µl of 1∶4,000 goat anti-human IgG-HRP conjugated antibody was added and incubated for 1 h at room temperature. The plates were then washed five times with 1× PBST. Finally, 100 µl of 3,3′,5,5′-tetramethylbenzidine substrate (Thermo Scientific, West Palm Beach, FL, USA) was added and the reaction was stopped with 3M H_2_SO_4_. The absorbance was read at 450 nm using ELISA reader (Tecan AG, Switzerland) and PBS was used as blank control.

### Statistical analysis

Statistical analyses were performed using SPSS version 17.0 (IBM, Armonk, NY, USA). Plasma levels of autoantibodies against HSP70, ENO1 and RNH1 proteins between patients groups were determined using student’s *t* test or non-parametric Mann-Whitney test. The correlation of HSP70 autoantibody level and disease progression across three groups, i.e. healthy controls, cholangitis and CCA, was analyzed by Spearman's rank correlation. The chi-square test was used to analyze the correlation between autoantibodies and the clinicopathological parameters. Diagnostic accuracy was assessed using the receiver operating characteristic (ROC) curve which was constructed by plotting the sensitivity versus (100%-specificity) and area under the curve (AUC) with 95% confidence intervals was calculated for each marker. *P* values lower than 0.05 were considered statistically significant.

## Results

### Identification of novel plasma autoantibodies

**Figure 1:**
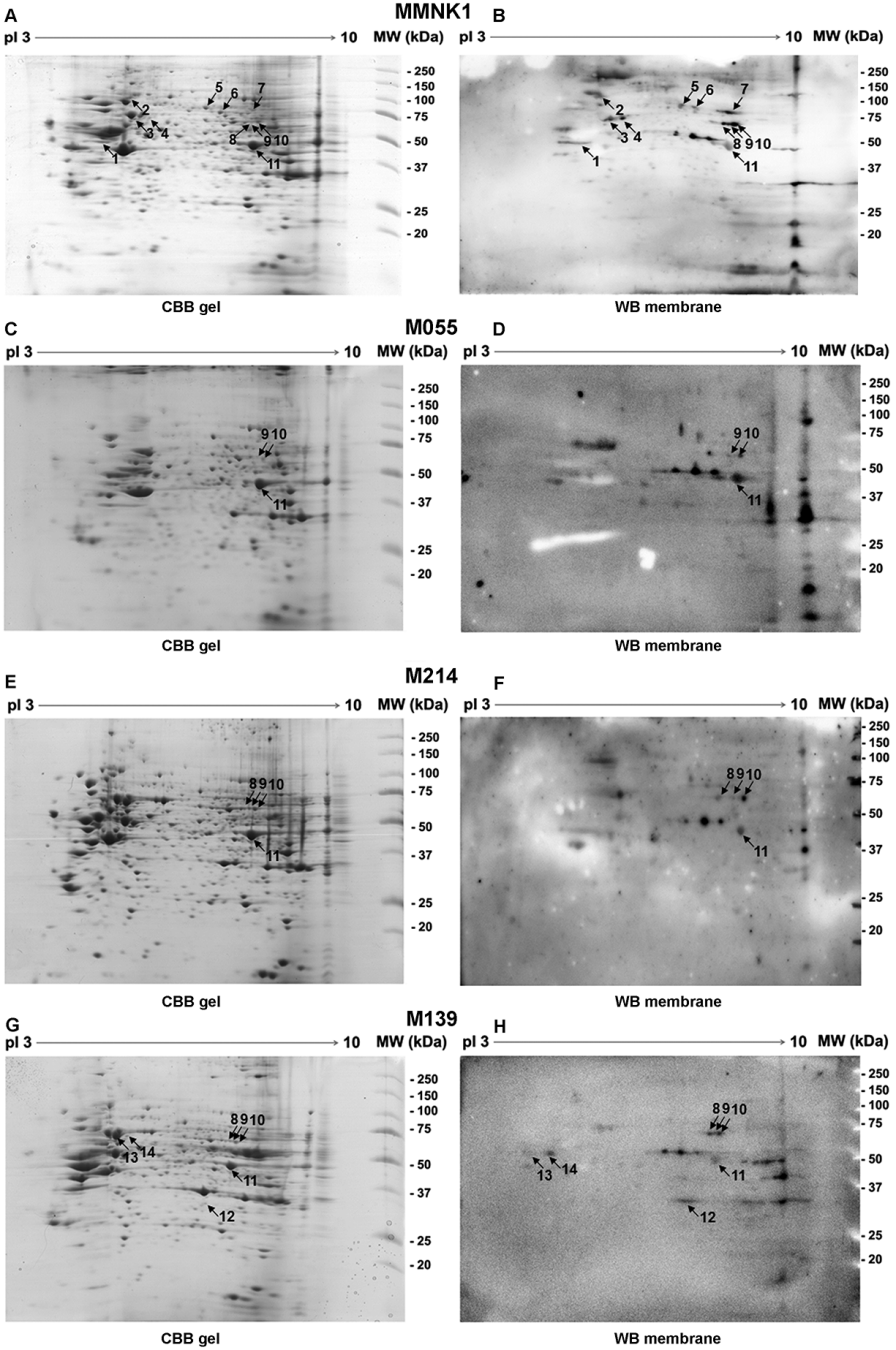
Two-dimensional (2D) gel electrophoresis and western blotting. MMNK1 and CCA cell lines (M055, M214 and M139) lysates were separated by 2D electrophoresis and stained with coomassie brilliant blue (panels A, C, E and G). Parallel gels were transferred to PVDF and incubated with pooled plasma of patients with CCA (n = 10) and incubated with secondary anti-human IgG (panels B, D, F and H). Matching IgG autoantibodies spots were numbered 1–14 in both CBB gels and western blot membranes. Duplicate experiments were performed. Spot numbers 1, 11 and 13 are RNH1, ENO1 and HSP70, respectively.

Eleven immunoreactive spots (spots No. 1–11) were successfully matched with MMNK1 cell line-derived proteins (Figure 1A–1B). In addition, three (spots No. 9–11), four (spots No. 8–11) and seven (spots No. 8–14) immunoreactive spots were successfully matched with M055, M214 and M139 CCA cell lines-derived proteins, respectively (Figure 1C–1H). Among all matched immunoreactive protein spots, three were found to be shared in all cell lines (spots number 9–11) and one of them was identified as enolase 1. Identification of autoantibodies against MMNK1 (Table 1), M055 (Table 2), M214 (Table 3) and M139 (Table 4) cell lines-derived proteins including their GI number, biological process and protein score was performed. Immunoreactivity against ribonuclease/angiogenin inhibitor 1 (RNH1, spot No. 1 in MMNK1 cell line), enolase 1 (ENO1, spot No. 11 in all cell lines) and heat shock protein 70 (HSP70, spot No. 13 in M139 cell line), which showed statistically significant of matched proteins with the highest protein score from each cell lines, was observed.

**Table 1:** Identification of autoantigens reacting against plasma samples from patients with CCA using MMNK1 cell line-derived proteins.

SpotNo.	Protein name	Biologicalprocess	GI number	Protein score	Coverage (%)	MW (kDa)	P-value	Species
1	Ribonuclease/angiogenin inhibitor 1	Angiogenesis	gi|15029922	143	13	50.1	*P*<0.05	Homo sapiens
2	Titin	Cardiac muscledevelopment	gi|17066105	52	1	384.3	*P*<0.05	Homo sapiens
3	Leucine-rich PPR-motif containing protein	Transcription regulation	gi|801893	58	6	146.3	ns	Homo sapiens
4	MSTP132	Unknown	gi|33338088	47	13	7.2	*P*<0.05	Homo sapiens
5	T-plastin polypeptide	Calcium ion binding	gi|190028	49	4	64.2	*P*<0.05	Homo sapiens
6	Enoyl Coenzyme A hydratase 1	Lipid metabolism	gi|119577225	93	32	14.3	*P*<0.05	Homo sapiens
7	PRP38 pre-mRNA processing factor 38	mRNA processing	gi|33988609	123	53	15.4	*P*<0.05	Homo sapiens
8	Alternative protein IPO7	Unknown	gi|444738577	51	51	12.7	ns	Homo sapiens
9	STIP1 protein	Stress response	gi|73909112	53	15	68.6	ns	Homo sapiens
10	FLJ10996 variant protein	Unknown	gi|68533041	58	17	76.1	ns	Homo sapiens
11	Enolase 1 variant (Alpha-enolase)	Glycolysis, Transcription regulation	gi|62896593	482	32	47.4	*P*<0.05	Homo sapiens

**Table 2:** Identification of autoantigens reacting against plasma samples from patients with CCA using M055 cell line-derived proteins.

SpotNo.	Protein name	Biological process	GI number	Protein score	Coverage (%)	MW (kDa)	P-value	Species
9	Heterogeneous nuclearribonucleoprotein L	Transcription regulation	gi|211828181	100	12	62.5	P<0.05	Homo sapiens
10	Unnamed protein product	Unknown	gi|10438562	57	23	36.6	P<0.05	Homo sapiens
11	Enolase 1 variant (Alpha-enolase)	Glycolysis, Transcription regulation	gi|62897945	672	65	47.5	P<0.05	Homo sapiens

**Table 3:** Identification of autoantigens reacting against plasma samples from patients with CCA using M214 cell line-derived proteins.

SpotNo.	Protein name	Biological process	GI number	Protein score	Coverage (%)	MW (kDa)	*P*-value	Species
8	ATP-dependent RNA helicase DDX3X	Apoptosis, Immunity, Transcription, translation regulation	gi|301171467	273	21	73.5	*P*<0.05	Homo sapiens
9	Heterogeneous nuclear ribonucleoprotein L	Transcription regulation	gi|211828181	100	12	62.5	*P*<0.05	Homo sapiens
10	Unnamed protein product	Unknown	gi|10438562	57	23	36.6	*P*<0.05	Homo sapiens
11	Enolase 1 variant (Alpha-enolase)	Glycolysis, Transcription regulation	gi|62897945	672	65	47.5	*P*<0.05	Homo sapiens

**Table 4:** Identification of autoantigens reacting against plasma samples from patients with CCA using M139 cell line-derived proteins.

SpotNo.	Protein name	Biological process	GI number	Protein score	Coverage (%)	MW (kDa)	*P*-value	Species
8	Unnamed protein product	Unknown	gi|194379588	50	11	52.8	*P*<0.05	Homo sapiens
9	Heterogeneous nuclear ribonucleoprotein L	Transcription regulation	gi|211828181	102	10	62.5	*P*<0.05	Homo sapiens
10	MSTP023	Transcription regulation	gi|17432233	79	25	26.3	*P*<0.05	Homo sapiens
11	Enolase 1 variant (Alpha-enolase)	Glycolysis, Transcription regulation	gi|62897945	818	50	47.5	*P*<0.05	Homo sapiens
12	Enoyl Coenzyme A hydratase 1	Lipid metabolism	gi|119577225	93	32	14.3	*P*<0.05	Homo sapiens
13	HSP70–1	Stress response	gi|4529893	701	27	70.2	*P*<0.05	Homo sapiens
14	T-plastin polypeptide	Calcium ion binding	gi|190028	49	4	64.2	*P*<0.05	Homo sapiens

### Plasma HSP70, ENO1 and RNH1 autoantibodies

**Figure 2:**
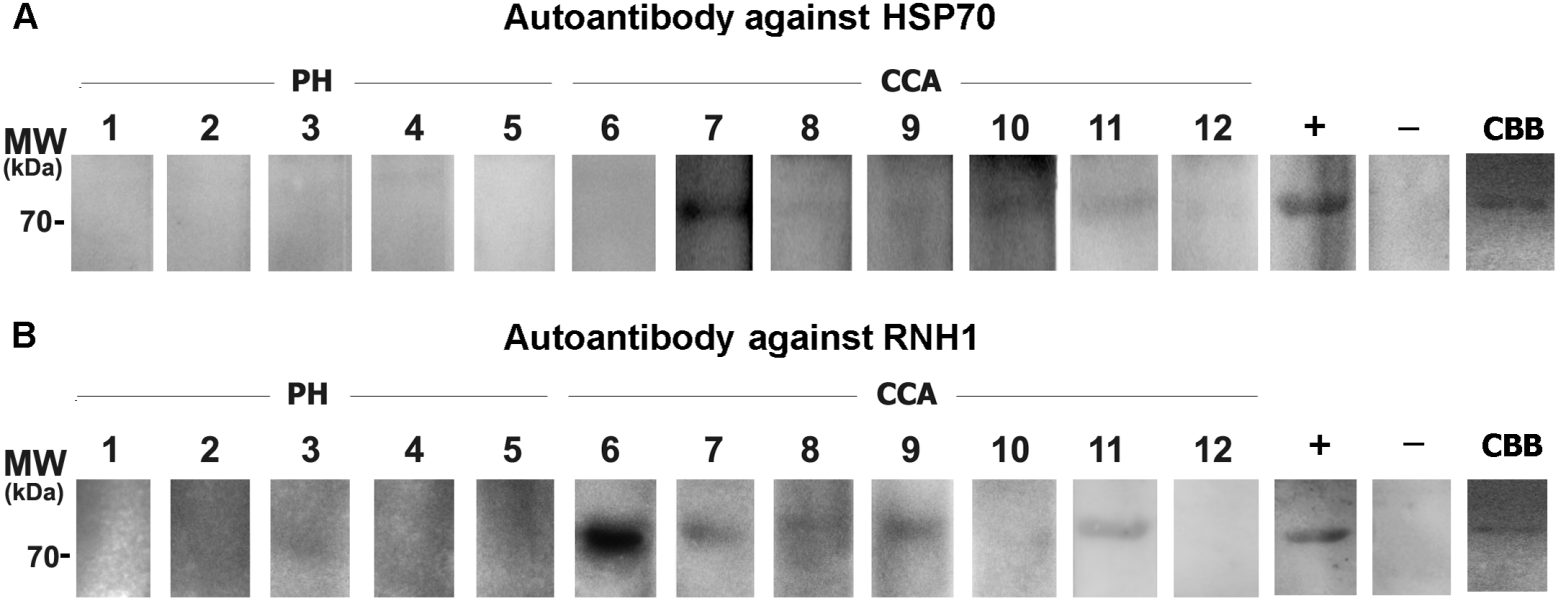
Western blotting analysis of HSP70 and RNH1 autoantibodies. Panel A) Recombinant human HSP70 was incubated with plasma of healthy individuals (lane 1–5) and patients with CCA (lanes 6–12). Immunoreactive bands were detected in 5/7 patients and none of the healthy controls. Panel B) Recombinant human RNH1 was incubated with plasma of healthy individuals (lane 1–5) and patients with CCA (lanes 6–12). Immunoreactive bands were detected in 6/7 patients and was weak in controls. Recombinant HSP70 and RNH1 proteins were probed with pooled healthy plasma served as negative controls (−) and pooled plasma from patients with CCA served as positive controls (+). HSP70 and RNH1 proteins were also demonstrated by staining with coomassie brilliant blue (CBB). PH  =  healthy individuals, CCA  =  cholangiocarcinoma.

Recombinant HSP70, ENO1 and RNH1 proteins were used as antigens and then probed with the plasma of CCA and healthy individuals using western blot. The 70 kDa immunoreactive band corresponding to HSP70 was reactive with 5/7 CCA but none of the healthy individual (Figure 2A) plasma samples. RNH1 autoantibody was detected in 6/7 CCA plasma and was weak in controls as a 76 kDa immunoreactive band (Figure 2B). None of the studied plasma manifested ENO1 autoantibody at western blot (*data not shown*) but confirmed its reactivity at ELISA (see below).

**Figure 3:**
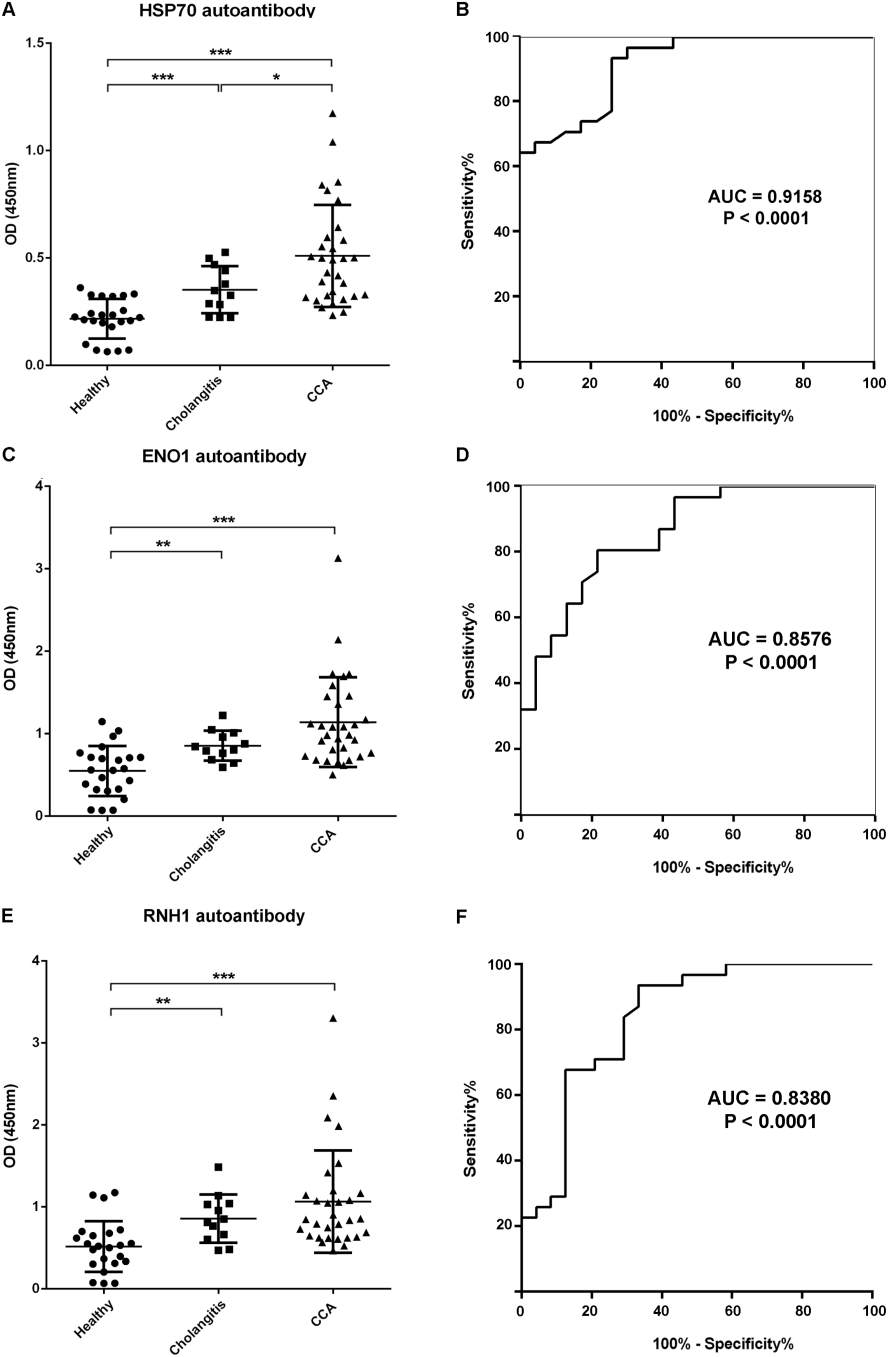
Plasma levels of HSP70, ENO1 and RNH1 autoantibodies by ELISA. Plasma levels of autoantibodies against HSP70, ENO1 and RNH1 were determined in clinical groups including cholangitis (n = 12), CCA (n = 31) and healthy controls (n = 23). Panel A) HSP70 autoantibody level is significantly higher in CCA compared to cholangitis and healthy controls. Panel B) Receiver operating characteristic (ROC) curves of HSP70 autoantibody in patients with CCA *vs* healthy controls. Panel C) ENO1 and Panel E) RNH1 autoantibodies are significantly higher in CCA than healthy controls. Panels D and F) ROC curves of ENO1 and RNH1 autoantibodies in patients with CCA *vs* healthy controls. Data are illustrated as mean ± SD using student’s *t* test or non-parametric Mann-Whitney test; *P<0.05, **P<0.01, ***P<0.001.

Using ELISA to test reactivities against all three antigens, the mean OD of blank, negative and positive controls were 0.045±0.02, 0.217±0.01 and 0.510±0.04 for anti-HSP70, were 0.056±0.02, 0.549±0.06 and 1.140±0.1 for anti-RNH1 and were 0.053±0.01, 0.525±0.06 and 1.063±0.11 for anti-ENO1, respectively. The highest titers of HSP70 autoantibody were found in CCA compared to cholangitis (*P*<0.05) and healthy individuals group (*P*<0.001, Figure 3A). Interestingly, when correlation of HSP70 autoantibody level and disease was analyzed across healthy controls, cholangitis and CCA group using Spearman's rank correlation, HSP70 autoantibody levels manifested a significant trend (r = 0.679, *P*<0.001). ENO1 autoantibody was undetectable by western blotting but was successfully demonstrated by ELISA. Plasma levels of ENO1 (Figure 3C) and RNH1 (Figure 3E) autoantibodies in cholangitis were significantly higher compared to healthy individuals (*P*<0.01). A statistically significant increase of ENO1 (*P*<0.001) and RNH1 (*P*<0.001) autoantibodies in CCA were observed compared to healthy individuals. Further, plasma ENO1 and RNH1 autoantibodies were higher in CCA compared to cholangitis but failed to reach statistical significance.

### Sensitivity and specificity of HSP70, ENO1 and RNH1 autoantibodies

The sensitivity and specificity of anti-HSP70, ENO1 and RNH1 autoantibodies are illustrated in Table 5. To discriminate patients with CCA from healthy controls, an anti-HSP70 cut-off value of 0.2630 yielded a sensitivity of 93.55% and a specificity of 73.91% (Table 5) with AUC of 0.9158 (Figure 3B). The positive predictive value was 82.86% and the negative predictive value was 89.47%. In the case of ENO1 autoantibody, the cut-off value of 0.7185 allowed a sensitivity of 80.65% and specificity of 78.26% (Table 5) with AUC of 0.8576 (Figure 3D) with a positive predictive value of 83.33% and a negative predictive value of 75.00%. For the RNH1 autoantibody, a cut-off value at 0.5615 had a sensitivity of 93.55% and a specificity of 78.26% (Table 5) with AUC of 0.8380 (Figure 3F). The positive predictive and negative predictive values were 85.29% and 90.00%, respectively.

**Table 5:** Diagnostic performance of autoantibodies against HSP70, RNH1 and ENO1 in pairwise comparisons between CCA, cholangitis, or healthy controls.

Diagnosis	AUC	Cutoff (OD)	Sensitivity (%)	95%CI	Specificity (%)	95% CI	*P*-value
Healthy *vs* CCA							
HSP70	0.9158	0.2630	93.55	78.58−99.21	73.91	51.60−89.77	<0.0001
ENO1	0.8576	0.7185	80.65	62.53−92.55	78.26	56.30−92.54	<0.0001
RNH1	0.8380	0.5615	93.55	78.58−99.21	66.67	44.68−84.37	<0.0001
Healthy *vs* cholangitis							
HSP70	0.8170	0.2695	75	42.81−94.51	73.91	51.60−89.77	0.002
ENO1	0.8043	0.7380	75	42.81−94.51	78.26	56.30−92.54	0.003
RNH1	0.7790	0.5785	83.33	51.59−97.91	65.22	42.73−83.62	0.007
Cholangitis *vs* CCA							
HSP70	0.7191	0.4800	51.61	33.06−69.85	83.33	51.59−97.91	0.02

AUC  =  area under the curve, 95%CI = 95% confidence interval (lower-upper).

Anti-HSP70, ENO1 and RNH1 autoantibodies levels could be distinguished between healthy controls and cholangitis (*P*<0.01, AUC = 0.8170, 0.8043 and 0.7790, respectively). The sensitivity and specificity rates of anti-HSP70 were 75% and 73.91%, of anti-ENO1 75% and 78.26% and of anti-RNH1 83.33% and 65.22%, respectively (Table 5). In addition, to discriminate between cholangitis and CCA the sensitivity and specificity of anti-HSP70 were 51.61% and 83.33%, respectively (*P*<0.05, AUC = 0.7191).

The discriminating power of the possible combinations of different reactivities of autoantibodies (i.e. HSP70 and ENO1, HSP70 and RNH1, ENO1 and RNH1, HSP70 and ENO1 and RNH1) was analyzed (Table 6) and the specificity exceeded 78% when compared to a single marker (Table 5) with only a slight reduction in sensitivity.

**Table 6:** Diagnostic performance (sensitivity, specificity, predictive values) of the possible combination of autoantibodies against HSP70, RNH1 and ENO1 in patients with CCA *vs* healthy controls.

Combination	Sensitivity (%)	Specificity (%)	PPV (%)	NPV (%)
HSP70+ENO1	77.42	82.61	85.71	73.08
HSP70+RNH1	87.10	82.61	87.10	82.61
RNH1+ENO1	74.19	78.26	82.14	69.23
HSP70+ENO1+RNH1	70.97	82.61	84.62	67.86

PPV  =  positive predictive value, NPV  =  negative predictive value.

### Clinical correlation of HSP70, ENO1 and RNH1 autoantibodies

The correlation between HSP70, RNH1 and ENO1 autoantibodies with the patient clinicopathological parameters is illustrated in Table 7. Also in consideration of the high baseline OD values observed with negative controls, OD levels of autoantibodies against HSP70, RNH1 and ENO1 were arbitrarily arrayed into high (OD>0.2630 for anti-HSP70, OD>0.7185 for anti-ENO1 and OD>0.5615 for anti-RNH1) or low (OD≤0.2630 for anti-HSP70, OD≤0.7185 for anti-ENO1 and OD≤0.5615 for anti-RNH1). Remarkably, the comparison between the CCA invasive phenotype and carcinoma *in situ* or mucinous type, identified elevated HSP70 and ENO1 autoantibodies as significantly correlated with CCA invasiveness (7 cases had high OD *vs* 5 cases had low OD of autoantibodies for invasive type and 6 cases had low OD *vs* no cases with high OD of autoantibodies for carcinoma *in situ* or mucinous type, *P* = 0.038 by chi-square test). Nonetheless, no correlation between autoantibodies levels and age, sex, histopathologic features, metastasis, or survival were seen (*data not shown*).

**Table 7:** Clinical and pathological features associated with HSP70, ENO1 and RNH1 autoantibodies in patients with CCA.

Variables	Anti-HSP70			*P*-value	Anti-ENO1			*P*-value	Anti-RNH1			*P*-value
	Low	High	Total		Low	High	Total		Low	High	Total	
Age (y)				0.87				0.623				0.576
≤56	8	9	17		7	10	17		9	8	17	
>56	7	7	14		7	7	14		6	8	14	
Gender				1.00				0.441				0.458
Male	10	11	21		8	13	21		9	12	21	
Female	5	5	10		6	4	10		6	4	10	
Histopathologic features				0.87				0.224				0.376
Tubular type	8	9	17		6	11	17		7	10	17	
Papillary type	7	7	14		8	6	14		8	6	14	
Tumor characteristics				0.038				0.038				0.152
Carcinoma in situ or mucinous type	6	0	6		6	0	6		5	1	6	
Invasive type	5	7	12		5	7	12		7	5	12	
Metastasis				0.685				0.671				0.685
Absent	4	3	7		4	3	7		4	3	7	
Present	11	13	24		10	14	24		11	13	24	

*When the sum of subset numbers does not match patient totals, data were missing or unavailable.

## Discussion

Post-translational modification of self-proteins can lead to aberrant protein expression or neo-antigens which may then cause the appearance of circulating autoantibodies [28]–[30], as observed in CCA [34]. We hypothesized that excess of modified proteins may contribute to induce autoantibodies in CCA and report for the first time that IgG autoantibodies against HSP70, ENO1 and RNH1 are specific to CCA and may prove candidates for challenging cases.

Among CCA-associated autoantigens identified in our study (Table 1–4), HSP70 protein is possibly the most intriguing because this is a stress response protein involved in various cell processes such as folding, assembly of newly synthesized proteins and the inhibition of apoptosis acting on the caspase-dependent pathway [35]. Overproduction of HSP70 leads to increased resistance against apoptosis-inducing agents such as tumor necrosis factor-α and doxorubicin [36] and can promote tumor growth and metastatic potential in rodent models [37]. Expression of HSP70 was found in CCA metastasis tissues [38] and could be used as marker in CCA [39]. Moreover, autoantibodies against HSP70 have been identified in esophageal squamous cell carcinoma, lung disease and alcohol-related disease [40]–[42] but not in CCA where elevated levels of carbonylated HSP70 protein were reported [34]. Our ELISA data support the view that autoantibodies against HSP70 may discriminate CCA from cholangitis, a risk condition for bile duct cancer, cholangitis from healthy individuals and CCA from healthy individuals (Table 5). Its level increased from healthy controls to cholangitis to CCA, suggesting that HSP70 autoantibodies might be used as not only for early marker but also for screening risk marker of CCA. High sensitivity (93.55%) and specificity (73.91%) of the test to discriminate between CCA from healthy individuals suggest that HSP70 autoantibodies might serve as a novel marker for CCA in addition to commonly used markers such as CA 19-9 (sensitivity 66% and specificity of 97%) [43] and CEA (sensitivity 68.0% and specificity of 81.5%) [44] which may, however, manifest similar performances for cancers other than CCA [13], [45], [46]. Also, IgG autoantibodies to HSP70 had higher sensitivity (93.55%) than protein level (80%) for CCA diagnosis [39]. In this case, HSP70 autoantibody level was significantly higher in CCA compared to cholangitis (*P*<0.05). Plasma HSP70 autoantibody level was positively correlated with the diseases progression from healthy control to cholangitis to CCA groups (r = 0.679, *P*<0.001), suggesting that its level correlates with the neoplastic potential and might be used as early marker of CCA. Interestingly, the specificity of HSP70 autoantibody levels in CCA was improved when combined with autoantibodies against ENO1 or RNH1 (Table 6). Therefore, using multiple autoantibodies rather than single marker may enhance CCA detection.

We demonstrated that plasma ENO1 autoantibody levels are also significantly increased in CCA and cholangitis compared to healthy control. ENO1, a multifunctional protein, is a glycolytic enzyme which is highly expressed in liver tissue and cytoplasm of hyperplastic bile ducts and overexpressed in different cancer types [47]. This protein is crucial to the response to hypoxia by tumor cell by increasing cell anaerobic metabolism [48] and its post-translational modifications by acetylation, methylation and phosphorylation could also be used for diagnostic and prognostic value in oncology [47]. Moreover, ENO1 is found on the surface of CCA cells and its overexpression is associated with poor prognosis and tumor invasiveness [49]. Western blot failed to identify ENO1 autoantibody in our series and we speculate that this may due to the loss of antigenicity of this protein during protein denature process for SDS-PAGE and the positive ELISA results support this view. Further, autoantibodies against RNH1 proteins were identified in CCA. RNH1 regulates the localization and activity of angiogenin which is involved in cell growth and survival mechanisms [50] as well as in cancer establishment, growth and metastasis [51]. RNH1 regulates the reactive oxygen species contribution to drug resistance in gastric cancer [52] and the growth and metastasis of bladder cancer [53].

CCA frequently manifests heterogeneous characteristics which might produce the different level of autoantibodies production. Our results demonstrate that circulating HSP70, ENO1and RNH1 autoantibodies levels are highest in CCA with a wide variability. Absorbance values in CCA group are high in some cases in parallel with elevated OD values in negative controls. In addition, although autoantibodies against HSP70, ENO1 and RNH1 proteins were not significantly associated with histopathologic features, metastasis, or survival, plasma HSP70 and ENO1 autoantibodies levels were able to discriminate between carcinoma *in situ* or mucinous from invasive phenotype of CCA. These findings are in agreement with the observation of autoantibody production in *Helicobacter pylori* infection-associated gastric cancer [15].

In conclusion, our data from a proteomic approach to a well-defined clinical cohort of patients demonstrate that CCA plasma include several IgG autoantibodies against host proteins and TAAs which are described herein for the first time. Higher level of HSP70, RNH1 and ENO1 autoantibodies are observed in CCA but lower level in healthy individuals or in patients with conditions that pose a clinical challenge to the physician. Underlying mechanisms remain unclear and warrant further studies. In particular, the combination of HSP70, ENO1 and RNH1 autoantibodies reactivities represent a good candidates as a new biomarker for CCA and should thus be investigated in a large-scale prospective study in high-risk subjects such as liver fluke infected Thai subjects.
